# *Bifidobacterium infantis* modulates intestinal microecology to inhibit the spread of antimicrobial resistance

**DOI:** 10.1128/msystems.00728-25

**Published:** 2025-10-31

**Authors:** Ziyun Li, Jiamin Hu, Yingmiao Pan, Yaxuan Xi, Lei Zhang

**Affiliations:** 1 Microbiome-X, School of Public Health, Cheeloo College of Medicine, Shandong University 12589, Jinan, China; 2 Shandong Provincial Maternal and Child Health Care Hospital Affiliated to Qingdao University, Qingdao University 371973 , Jinan, China; 3 State Key Laboratory of Microbial Technology, Shandong University 520252, Qingdao, China; California State University Stanislaus, Turlock, California, USA

**Keywords:** *Bifidobacterium infantis*, antimicrobial resistance, bile acids, horizontal gene transfer, gut microbiota

## Abstract

Early administration of antibiotics in children may heighten the susceptibility to multidrug-resistant bacterial infections. While probiotics are commonly employed for bacterial infection management, their nuanced advantages, particularly in curtailing the spread of antimicrobial resistance (AMR), remain unclear. This study investigated the role and mechanisms of *Bifidobacterium infantis* in inhibiting the spread of antibiotic resistance genes (ARGs) in the gut. We found that supplementing with *B. infantis* 15697 significantly enhanced the synthesis of bile acids in mouse feces, particularly tauroursodeoxycholic acid (TUDCA) and taurocholic acid (TCA). Concurrently, the abundance of potential probiotics such as *Parabacteroides goldsteinii* in the gut significantly increased. Using a mouse infection model, we discovered that *B. infantis* supplementation inhibited the colonization of antibiotic-resistant *Escherichia coli* in the gut and the events of horizontal gene transfer, thereby reducing the spread of ARGs. Further analysis revealed that TUDCA and TCA, through their interaction with the OmpC protein, decreased the biofilm formation capability and cell membrane permeability of antibiotic-resistant bacteria, inhibiting the horizontal spread of ARGs. These findings reveal the important role of *B. infantis* in regulating the gut microbiota and inhibiting the spread of ARGs, providing a theoretical basis for developing new probiotic intervention strategies. This could help reduce the global spread of AMR and protect human health.

**IMPORTANCE:**

The global spread of antimicrobial resistance (AMR) has become a significant threat to public health, particularly in children, where the overuse of antibiotics leads to gut microbiota imbalance and increases the risk of horizontal transfer of antibiotic resistance genes (ARGs). This study supplemented mice with *Bifidobacterium infantis* 15697, which significantly enhanced the synthesis of bile acids, especially tauroursodeoxycholic acid and taurocholic acid, while promoting the growth of probiotics and inhibiting the colonization of antibiotic-resistant bacteria and the spread of ARGs. This finding not only reveals the important role of *B. infantis* in regulating the gut microbiota and inhibiting the spread of ARGs but also provides a theoretical basis for developing new probiotic intervention strategies. By modulating the gut microbiota and bile acid metabolism, *B. infantis* has the potential to become an effective means of reducing the spread of AMR. This is of great significance for protecting the gut health of children and adults, reducing the risk of resistant infections, and also provides scientific evidence for the formulation of global public health policies.

## INTRODUCTION

Antimicrobial resistance (AMR) is an ancient and insidious epidemic that has emerged as a significant threat to global public health (1, 2). It is estimated that in 2021, there were 4.71 million deaths globally associated with bacterial AMR, of which 1.14 million deaths were directly attributable to bacterial AMR (3). It is predicted that by 2050, more than 39 million people will die from antibiotic-resistant infections (4). Globally, approximately 200,000 neonatal deaths are attributed to AMR annually (5, 6). The overuse and misuse of antibiotics are the primary drivers of AMR dissemination. The majority of children worldwide begin antibiotic treatment before the age of 2, particularly in low-income and middle-income countries (7–9). The first 1,000 days of life are critical for the formation of an infant’s gut microbiota; however, children in this stage often receive a higher proportion of unnecessary broad-spectrum antibiotic treatments (10–12), leading to the eradication of beneficial bacteria, such as *Bifidobacterium infantis*, and the disruption of the gut microbiome (13).

A 2007 study first demonstrated through *in vitro* experiments that *Bifidobacterium* inhibits the horizontal transfer of antibiotic resistance genes (ARGs) among Enterobacteriaceae by suppressing plasmid-mediated AMR dissemination (14, 15). Subsequent omics analyses in 2018 (16) and 2023 (17) revealed an inverse correlation between high *Bifidobacterium* abundance and reduced ARG richness within infant gut microbiomes, though the underlying mechanisms remain unclear. These findings suggest that *Bifidobacterium* may directly interfere with the horizontal dissemination of AMR through specific molecular pathways. Infants and young children are particularly susceptible to antibiotic-resistant bacteria due to their dynamically developing gut microbiota and immature intestinal epithelial tight junctions, which heighten bacterial-host interactions (18). This vulnerability is compounded by the frequent administration of broad-spectrum antibiotics—often unnecessarily prescribed at high rates before age 2 (8, 9, 11, 12). Such antibiotic exposure reduces *Bifidobacterium* colonization while enriching mobile ARGs (mARGs), thereby elevating risks of severe infections in this population (18, 19).

Plasmid-mediated horizontal transfer of ARGs represents a primary route for the evolution of pathogen resistance (20–29). Conjugative plasmids carrying carbapenemase resistance genes are notably prevalent in infant gut microbiomes (30). Our previous study demonstrated that co-localization of mARGs with mobile genetic elements (MGEs) is widespread in Enterobacteriaceae isolated from infant intestines (31), indicating that the infant gut harbors abundant mARGs with high transmission potential. Critically, if these mARGs integrate into conjugative plasmids, pathogens or opportunistic pathogens acquiring such plasmids could rapidly evolve into multidrug-resistant (MDR) strains. Moreover, studies have shown that when antibiotic-resistant pathogens invade the host and cause infections, their resistance plasmids can stably replicate in resident gut bacteria through conjugation even after the pathogens are eliminated (25). The co-localization of mARGs with MGEs further facilitates the sharing of resistance genes across multiple compatible plasmids within bacterial populations (26). Alarmingly, the World Health Organization has emphasized that none of the currently approved or investigational antibiotics are effective in curbing the growth and spread of AMR (32). In the context of limited pediatric medication options, neonates, especially preterm and extremely preterm infants, may face the burden of untreatable MDR bacterial infections, imposing significant costs on families and society (33, 34). Therefore, investigating the relationship between *Bifidobacterium* and AMR dissemination among gut bacteria, along with its underlying mechanisms, holds critical significance for ensuring long-term health outcomes in children.

Evidence indicates that infancy is the life stage with the highest abundance of *Bifidobacterium* in the human gut, particularly *B. infantis*, which rapidly decreases and may be undetected by the age of 2–3 years (35). We hypothesize that supplementing with *B. infantis* could reduce the horizontal transfer of AMR among gut bacteria. To test this hypothesis, we conducted a series of experiments using both murine infection models and at the cellular level. The results demonstrated that although the supplemented *B. infantis* 15697 failed to persistently colonize healthy specific pathogen-free (SPF) mice and did not significantly alter the microbiome structure, it significantly enhanced the abundance of probiotics associated with diverse bile acid profiles in the gut. Furthermore, it facilitated bile acid synthesis and the levels of taurine-conjugated bile acids, such as taurocholic acid (TCA). Studies have shown that bile acids, including TCA, modulate intestinal epithelial cell proliferation (36), a process that may influence bacterial membrane permeability. This modulation could alter antibiotic uptake and efflux, thereby reducing bacterial antibiotic resistance. Furthermore, bile acids may inhibit biofilm formation by interfering with the cyclic diguanylate monophosphate signaling pathway (37, 38). Crucially, biofilm architecture and local bacterial density are key determinants of plasmid-mediated AMR dissemination (39). Both *in vitro* and *in vivo* results from this study demonstrate that the supplementation of *B. infantis* 15697 can inhibit the horizontal transfer of mARGs among bacteria. This effect may be mediated by increased fecal levels of tauroursodeoxycholic acid (TUDCA) and TCA, which inhibit the bacterial efflux porin OmpC, reduce membrane permeability, and lower local bacterial density—collectively diminishing conditions favorable for AMR dissemination.

## RESULTS

### Supplementation of *B. infantis* 15697 significantly enhances bile acid synthesis and the excretion of taurine-conjugated bile salts in feces

**Fig 1:**
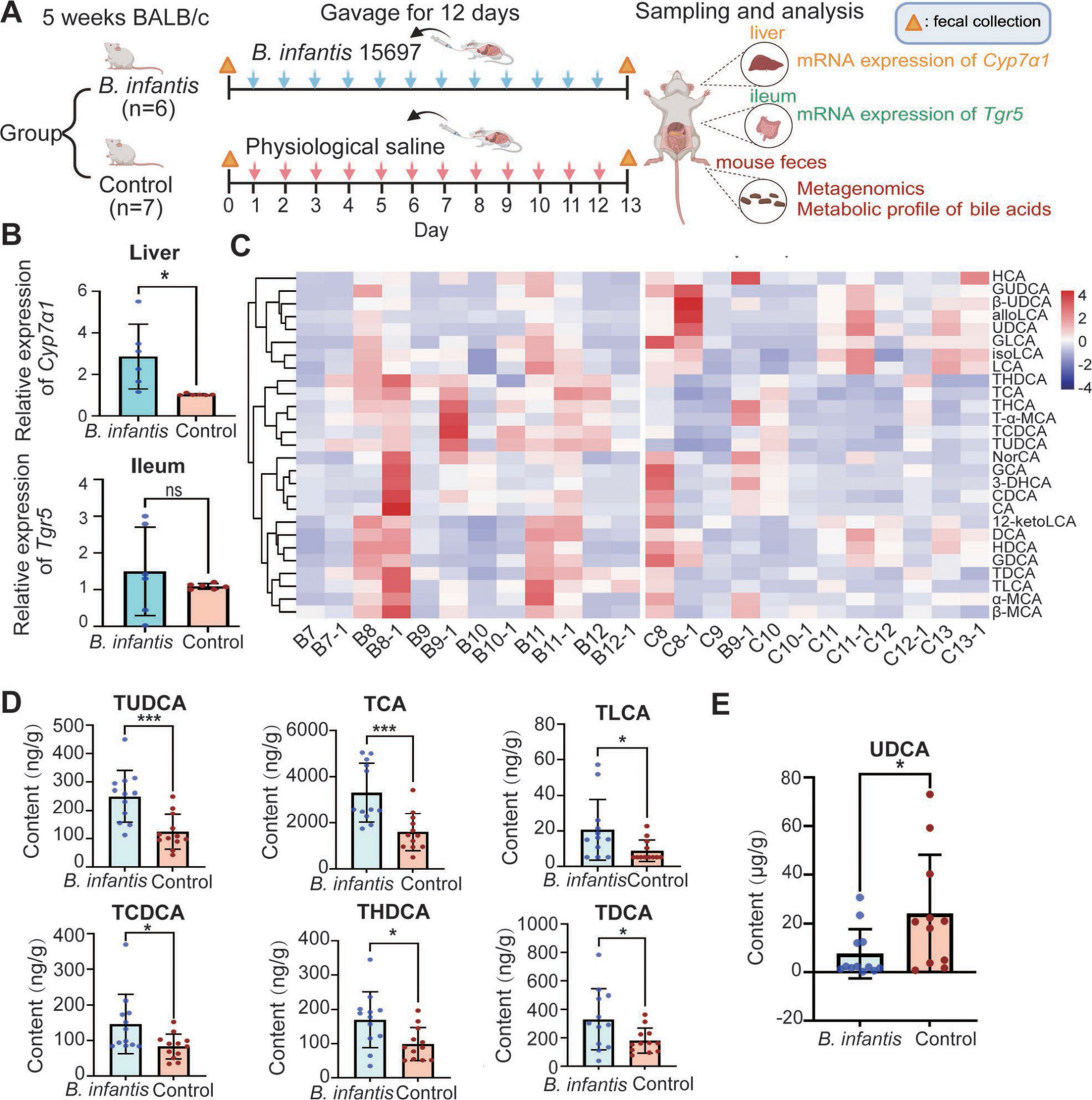
Comprehensive analysis of bile acid metabolism and gene expression in animal models**.** (**A**) Schematic diagram of the animal experimental procedure. (**B**) Expression levels of relevant genes in liver or ileum tissue. (**C**) Bile acids significantly increased in feces of the experimental group compared to the control group. Compared with the control group, the experimental group showed (**D**) a significant increase in six bile acids and (**E**) a significant decrease in UDCA content in feces. *B. infantis* group represents the condition after continuous gavage with *B. infantis* 15697 for 12 days. Control in group represents the condition after continuous gavage with saline for 12 days. ***, *P* < 0.001; *, 0.01 < *P* < 0.05.

As depicted in Fig. 1A, we administered a 12-day gavage of *B. infantis* 15697 to healthy 5-week-old mice and examined the expression of genes associated with bile acid metabolism and inflammation in the liver and ileum after the continuous 12-day gavage (Fig. 1B; Fig. S1). The results revealed that, compared to the control group, supplementation with *B. infantis* significantly upregulated the expression of *Cyp7α1* in the livers of mice, which encodes the rate-limiting enzyme for bile acid synthesis (*P* < 0.05, Fig. 1B). However, the expression of the FXR (farnesoid X receptor, a core suppressor of bile acid synthesis) and TLR4 (toll-like receptor 4, which recognizes bacterial lipopolysaccharide to trigger inflammatory responses) encoding gene in the liver, as well as the *Tgr5* (Takeda G protein-coupled receptor 5, a receptor specifically binding bile acids) in the ileum, showed no significant changes compared to the control group (Fig. S1).

We subsequently performed a quantitative analysis of fecal bile acid metabolites. Although individual variations were observed—with mouse C8 in the control group and mouse B7 in the *B. infantis* group showing notably different bile acid profiles compared to their respective groups (Fig. 1C)—overall, supplementation with *B. infantis* 15697 was found to promote an increase in the levels of certain bile acids in feces (Fig. 1C and D; Table S1). Interestingly, compared to the control group, the experimental group showed a significant upregulation of bile acid salts conjugated with taurine (Fig. 1D), with the content of ursodeoxycholic acid (UDCA) being significantly reduced (Fig. 1E). These results suggest that *B. infantis* may enhance the biological activity of bile acids by promoting their biosynthesis and increasing the content of taurine-conjugated bile acids in feces.

### Supplementation of *B. infantis* 15697 significantly promotes the abundance of probiotic *Parabacteroides goldsteinii* in feces and reduces horizontal transfer of mARGs among bacteria

**Fig 2:**
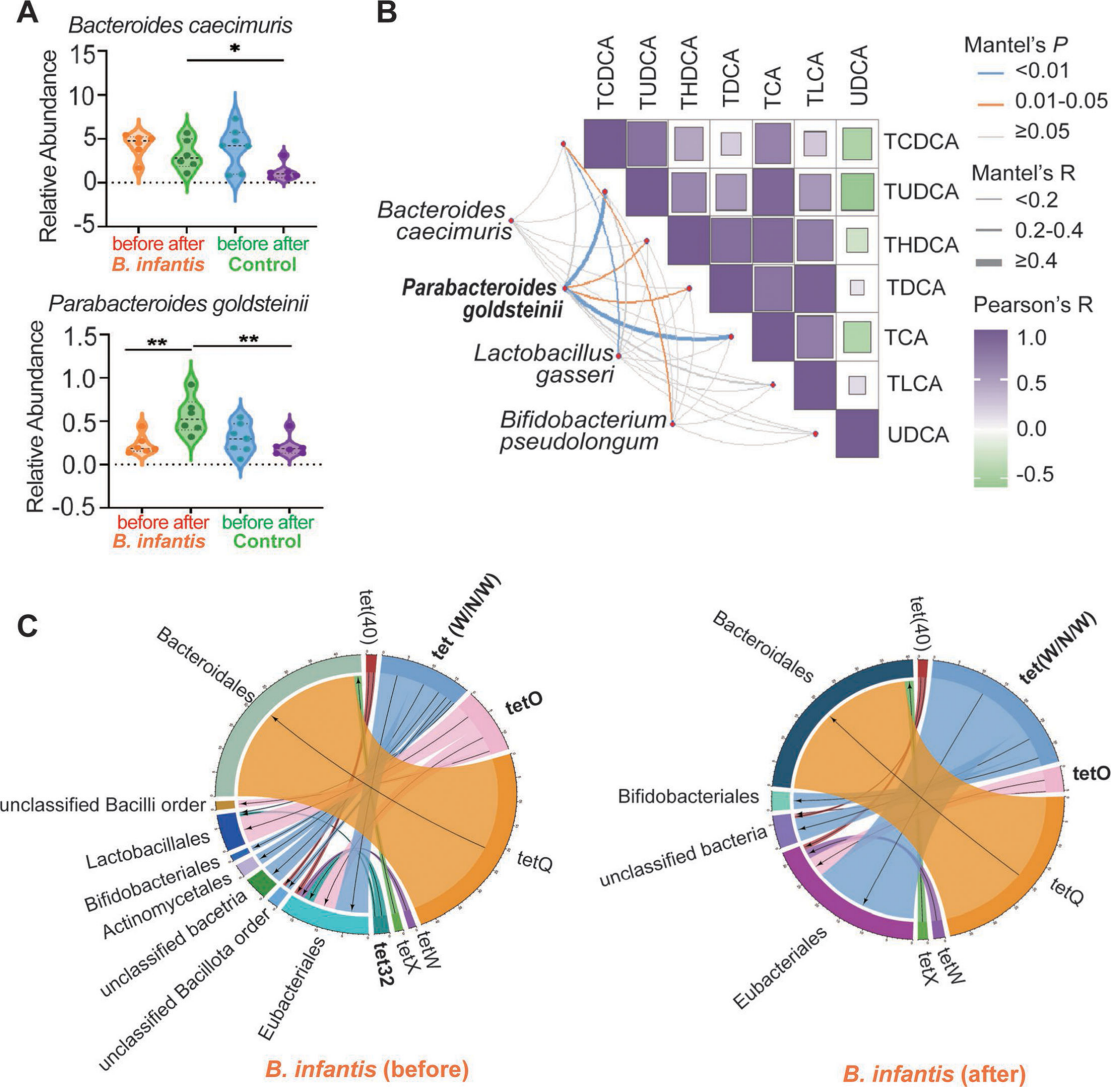
Effects of *B. infantis* 15697 gavages on the gut bacteria and ARGs in mice. (**A**) Relative abundance of significantly enriched strains in the *B. infantis* 15697 gavage group (with FDR-corrected *P* values). (**B**) Correlation analysis between four potential probiotics and the levels of significantly changed bile acids in feces. (**C**) The association analysis between strains and tetracycline resistance-associated mARGs before and after gavage in the *B. infantis* group. **, 0.001 < *P* < 0.01; *, 0.01 < *P* < 0.05.

Compared to the control group, supplementation with *B. infantis* 15697 did not significantly alter the α-diversity and β-diversity of the gut microbiota in mice (Fig. S2). Linear discriminant analysis effect size (LEfSe) analysis (without FDR correction) revealed that, compared to the control group, the abundance of potential probiotics, including *Bacteroides caecimuris*, *Parabacteroides goldsteinii*, *Lactobacillus gasseri*, and *Bifidobacterium pseudolongum*, showed increased abundance after supplementation with *B. infantis* 15697 (Fig. S3). Next, we performed differential species analyses using LEfSe (with FDR correction), ANCOM-BC (with Holm-Bonferroni correction), and MAasLin2 (with FDR correction). The results indicated that no significant differences were detected between the control and *B. infantis* groups, which is consistent with the non-significant changes in α-diversity and β-diversity. Significance analysis was performed for the four bacterial taxa with increased abundance in the *B. infantis* group, as shown in Fig. S3, revealing that only *B. caecimuris* and *P. goldsteinii* in the fecal microbiome of the *B. infantis* group exhibited statistically significant differences compared to the control group (Fig. 2A; Fig. S4, with FDR correction). This indicates no substantial divergence in microbiome composition between groups, despite significant structural changes in bile acid profiles. Notably, the abundance of *P. goldsteinii* was significantly positively correlated with the levels of various bile acids in feces, such as TUDCA, taurohyodeoxycholic acid (THDCA), taurodeoxycholic acid (TDCA), and TCA, with the highest correlation and significance observed for TUDCA and TCA (Fig. 2B; *P* < 0.01, *R* > 0.4). Additionally, we comprehensively analyzed the ARGs in the mouse gut and accurately linked them to their host strains to explore their potential inter-strain dissemination, following our previous methods (40). Before saline gavage in the control group, the vancomycin resistance gene *vanI* was only found in bacteria of the Erysipelotrichales, Eubacteriales, Kitasatosporales, and unclassified bacterial orders (Fig. S5). However, after 12 days of saline gavage, *vanI* was also detected in Bacillales and Mycobacteriales (Fig. S5), suggesting possible horizontal gene transfer (HGT) events between bacteria. Similarly, the linezolid resistance gene *poxtA* appeared to have entered Bacillales through possible HGT in the control group (Fig. S5). In contrast, after 12 days of gavage with *B. infantis* 15697, the abundance of ARG-carrying strains decreased, and ARGs were not detected in the order of Mycobacteriales, Actinomycetales, unclassified Bacillota, and unclassified Bacilli (Fig. S5).

Notably, focusing on tetracycline resistance-related mARGs, we found that after 12 days of *B. infantis* 15697 gavage, Eubacteriales carrying *tet32* were undetectable, and this effect extended to other species (Fig. 2C). Additionally, the number of strains carrying *tet(W/N/W*) and *tetO* also significantly decreased (Fig. 2C). In the control group, after 12 days of saline gavage, except for *tetW*-carrying unclassified bacteria, which were undetectable, other ARGs remained in their original strains (Fig. S6). This shows that gavage with *B. infantis* 15697 reduces mARGs dissemination of the inter-strain.

### Supplementation of *B. infantis* 15697 effectively suppresses the AMR dissemination in the mouse intestine

**Fig 3:**
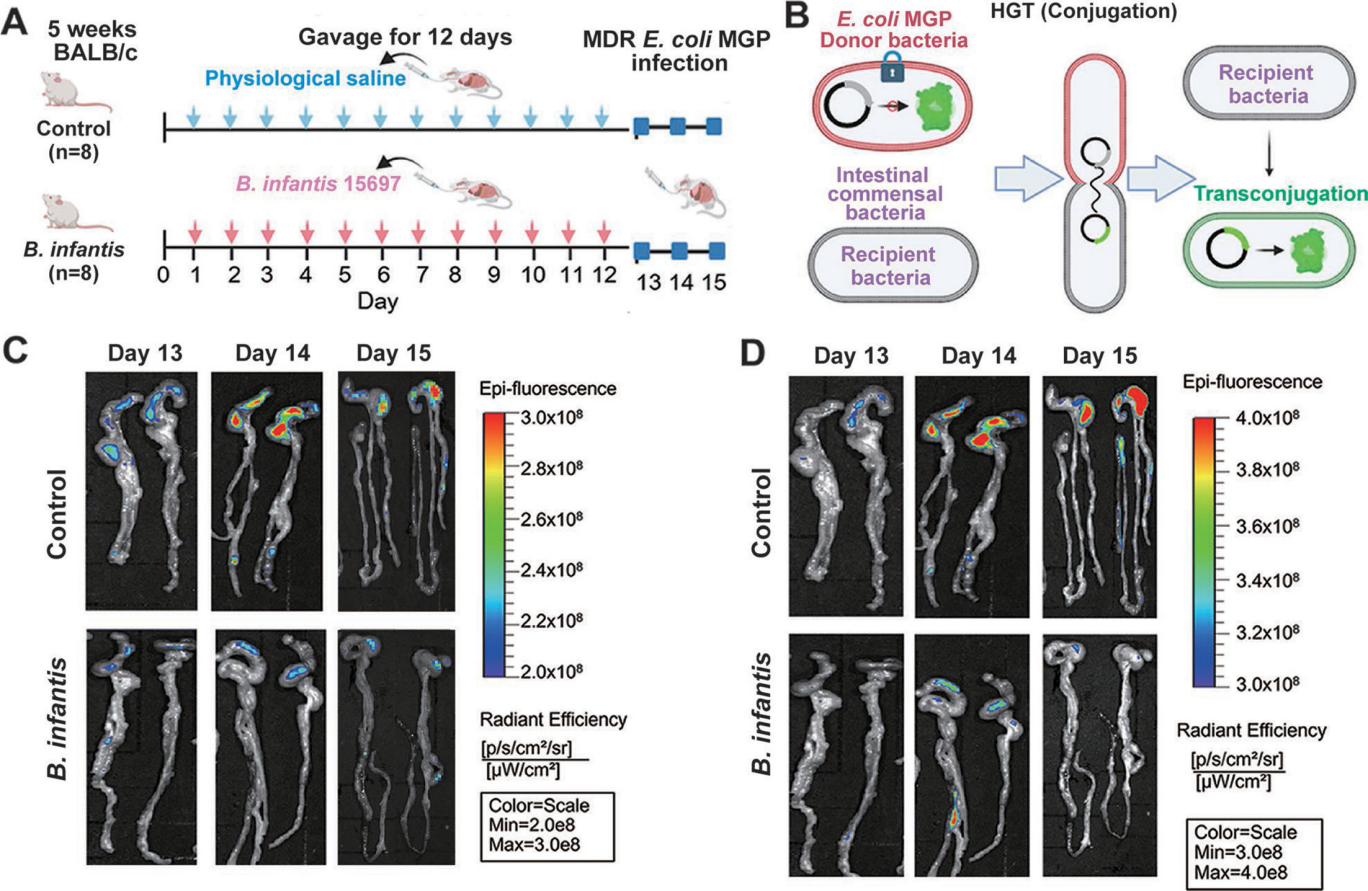
Monitoring plasmid-mediated HGT in the mouse gut using a dual-color fluorescence reporter system. (**A**) Schematic diagram of the mouse infection model. (**B**) Fluorescence expression diagram of the donor bacteria. The donor bacteria carry a plasmid encoding green fluorescent protein on their chromosome, which is restricted in expression, thus appearing red. When the plasmid is transferred to other gut commensal bacteria through conjugation, the restriction is lifted, and the transconjugants exhibit green fluorescence. (**C**) Red fluorescence intensity (indicating the colonization status of the donor *E. coli*). (**D**) Green fluorescence intensity (indicating the occurrence of HGT events).

To further clarify whether the supplementation of *B. infantis* reduces the reservoir of mARGs in gut commensal bacteria during the infection period of antibiotic-resistant bacteria in mice by inhibiting the spread of AMR levels, we designed a mouse infection experiment (Fig. 3A). The control group and the *B. infantis* gavage group were administered saline and *B. infantis* 15697, respectively, for 12 consecutive days, followed by infection with antibiotic-resistant *Escherichia coli* MGP from day 13 to 15. The antibiotic-resistant *E. coli* MG1655::*lacI^q^-pLpp-mCherry-Km*^R^ (pKJK5::*gfpmut3b*) (named *E. coli* MGP), carrying the IncP-1ɛ broad-host range plasmid pKJK5::*gfpmut3b*. The plasmid pKJK5::*gfpmut3b* does not express green fluorescence in *E. coli* MGP, so it only expresses red fluorescence. However, when pKJK5::*gfpmut3b* is transferred to other gut commensal bacteria through conjugation, these commensal bacteria express green fluorescence (Fig. 3B). Using the mouse infection model, we found that in the control group, after infection with *E. coli* MGP, the bacterium was able to colonize in the mouse gut (Fig. 3C) and spread AMR through HGT (Fig. 3D). However, compared to the control group, after 12 days of *B. infantis* gavage as a protective measure, the colonization of *E. coli* in the mouse gut was inhibited (Fig. 3C), and the occurrence of HGT spreading AMR was reduced (Fig. 3D).

**Fig 4:**
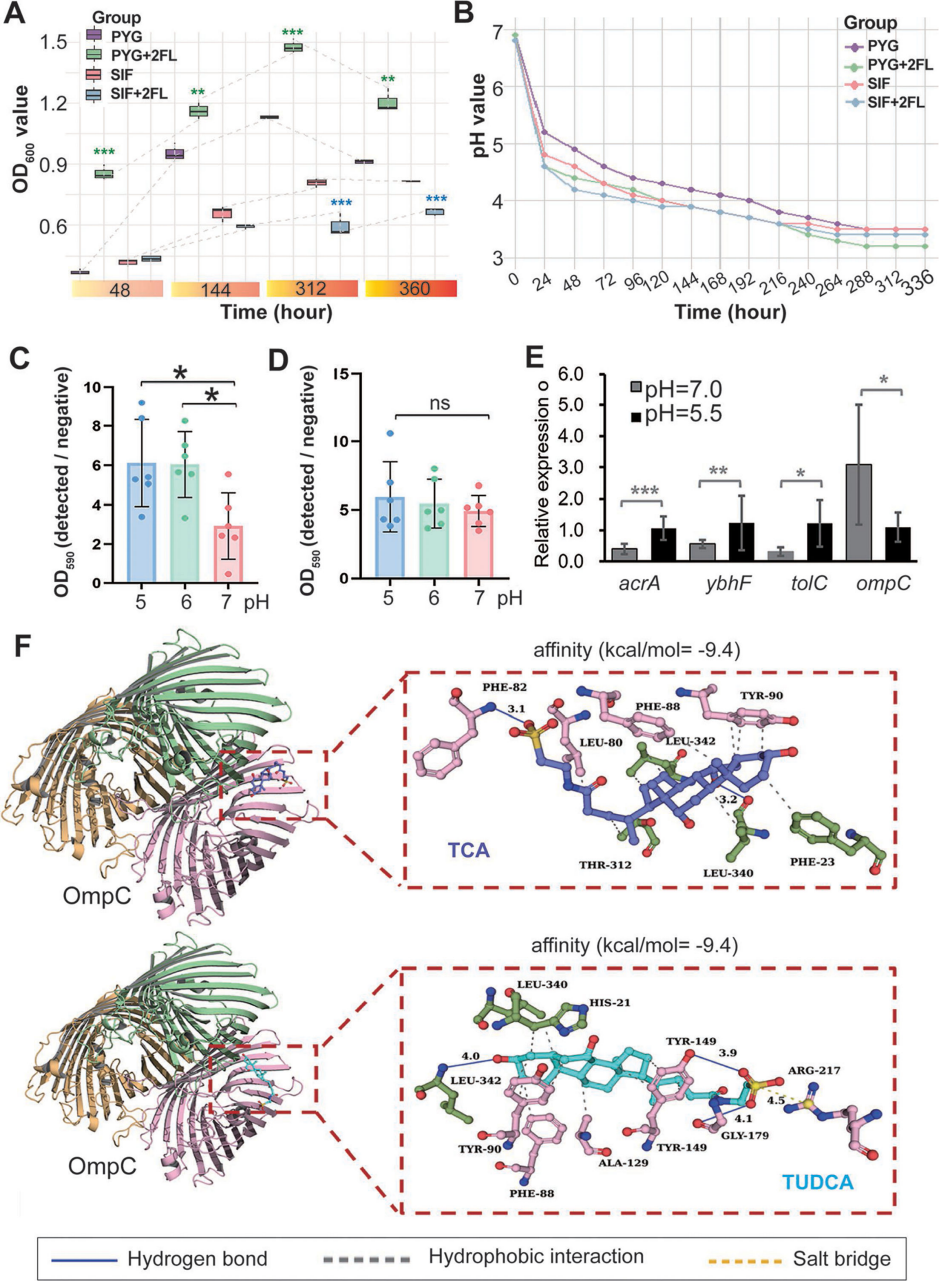
Multidimensional analysis of mouse infection model and mechanisms of AMR dissemination. (**A**) Effects of culture conditions and HMO on the *in vitro* growth of *B. infantis*. (**B**) The changes in pH levels of *B. infantis* cultures under different conditions and with or without the addition of HMO. **(C** and **D**) The biofilm formation of *E. coli* DH5α and DH5α (pRP4) under different pH conditions. (**E**) Gene expression in *E. coli* DH5α (pRP4). (**F**) Analysis of the interaction between TCA and TUDCA with the OmpC protein. Yellow, pink, and green represent the A, B, and C chains of OmpC, respectively. Purple and blue represent TCA and TUDCA, respectively. ***, P < 0.001; **, 0.001 < *P* < 0.01; *, 0.01 < *P* < 0.05.

Even after 12 consecutive days of *B. infantis* supplementation, long-term colonization was not observed. However, its transient survival may have facilitated the proliferation of other probiotics (*B. caecimuris* and *P. goldsteinii*) through temporary alterations in intestinal pH or local metabolic environments. To further investigate the effects of human milk oligosaccharides (HMO) (in this study, 2′-fucosyllactose, 2FL, was used) on *B. infantis* growth, *in vitro* culture experiments were conducted in either peptone yeast glucose (PYG) broth base medium or simulated intestinal fluid (SIF, pH = 6.8). In PYG medium, supplementation with 2FL significantly enhanced *B. infantis* growth (Fig. 4A) and contributed to a reduction in the overall pH of the culture metabolites (Fig. 4B). Intriguingly, in SIF, *B. infantis* growth was significantly reduced in the 2FL-supplemented group compared to the control (Fig. 4A), despite 2FL supplementation lowering the overall pH of the culture metabolites (Fig. 4B). This demonstrates that 2FL exerts a marked environment-dependent influence on the growth of *B. infantis* 15697: in nutrient-rich PYG medium, 2FL acts as a preferential carbon source to stimulate bacterial proliferation and amplify acidogenic metabolism, whereas in SIF, 2FL supplementation paradoxically suppresses biomass formation, indicating intricate regulatory mechanisms governing the strain’s metabolic plasticity and ecological adaptation within gut-mimetic environments.

Continuous supplementation with *B. infantis* promotes bile acid synthesis and increases the abundance of *P. goldsteinii*, which is significantly positively correlated with TUDCA and TCA (*R* > 0.4, *P* < 0.01). This helps to regulate the composition of bile acids in the gut, thereby further maintaining intestinal pH stability. To further assess pH-dependent biofilm regulation, we acidified the culture medium with acetic acid. Notably, pH perturbation exerted no detectable effect on biofilm formation in antibiotic-resistant *E. coli* DH5α (pRP4) (Fig. 4D). In contrast, maintaining neutral pH (7.0) significantly attenuated the biofilm formation in antibiotic-sensitive *E. coli* DH5α (*P* < 0.01, two-tailed *t*-test; Fig. 4C). Further analysis revealed that a lower pH significantly promoted the expression of multidrug efflux pumps and significantly reduced the expression of the OmpC porin-encoding gene (Fig. 4E), indicating that a lower pH is conducive to enhancing the antibiotic resistance of bacteria and aiding their colonization. However, the significant increase in bile salts in the gut, especially TUDCA and TCA, may promote the gut pH to be neutral or slightly alkaline, which will significantly reduce the expression of multidrug efflux pumps and OmpC porin in pathogenic bacteria, inhibiting the colonization of opportunistic pathogens; it will also significantly reduce the biofilm formation of antibiotic-sensitive opportunistic pathogens, thereby reducing the horizontal spread of AMR.

In this study, we further simulated the molecular docking of TUDCA and TCA, two bile salts, with the OmpC protein (Fig. 4F). The affinity score of TCA with OmpC protein was −9.4 kcal/mol, with TCA forming hydrogen bonds with PHE-82 and LEU-340 of the OmpC protein, and hydrophobic interactions with LEU-80, PHE-88, LEU-342, and TYR-90. The affinity score of TUDCA with OmpC protein was also −9.4 kcal/mol. TUDCA formed hydrogen bonds with LEU-342, GLY-179, and TYR-19 of the OmpC protein, a salt bridge with ARG-217, and hydrophobic interactions with LEU-340, HIS-21, TYR-90, PHE-88, ALA-129, and TYR-149 (Fig. 4F). Both bile salts can bind tightly to OmpC, suggesting that the supplementation of *B. infantis*, promoting the enrichment of TUDCA and TCA in the gut, may reduce the intestinal colonization of opportunistic pathogens and the spread of AMR between bacteria through binding with OmpC.

## DISCUSSION

This study employed integrated multi-omics approaches to elucidate a potential mechanism by which *B. infantis* 15697 modulates bile acid metabolism to suppress horizontal dissemination of AMR. The data demonstrated that although prolonged supplementation of this strain did not induce global restructuring of the gut microbiota (Shannon index: *P* > 0.05, Bray-Curtis dissimilarity: *P* = 0.066), it elicited functionally significant modulatory effects. Specifically, *B. infantis* 15697 administrations significantly upregulated hepatic Cyp7α1 gene expression (*P* < 0.05) and enhanced fecal accumulation of taurine-conjugated bile acids, particularly TUDCA (*P* < 0.001) and TCA (*P* < 0.001). Concurrently, we observed selective enrichment of *P. goldsteinii* (FDR < 0.01), a commensal species with established bile acid metabolic capabilities. Notably, the observed inhibition of AMR dissemination events in the murine gut following *B. infantis* supplementation appears minimally associated with microbial community restructuring. Instead, mechanistic insights suggest stronger correlations with compositional shifts in intestinal bile acids and reductions in local bacterial density (biofilm formation). This finding aligns with prior studies emphasizing microenvironmental modulation (particularly reducing the ratio of biofilms formed by newly proliferated bacteria to those formed by mature mother cells) over structural reorganization in AMR containment (39). The transient yet persistent ecological impact of *B. infantis* 15697 underscores its distinctive role as an “ecological engineer.” Despite its failure to achieve stable colonization, the strain induced sustained microenvironmental remodeling through transient metabolic activity. This transient colonization strategy, characterized by bile acid-mediated signaling and cross-feeding interactions, represents an underappreciated mechanism of probiotic functionality that warrants further investigation.

Bile salts play a pivotal role in the human digestive system, facilitating lipid digestion and absorption while modulating host health through interactions with the gut microbiota (41, 42). Our study demonstrates that *B. infantis* supplementation significantly upregulated hepatic expression of CYP7α1 (*P* < 0.05), the rate-limiting enzyme in bile acid synthesis, and increased fecal concentrations of TUDCA and TCA. These findings indicate that *B. infantis* administration reshapes the intestinal bile acid pool composition. TUDCA and TCA, two critical bile salts, enhance lipid emulsification and nutrient absorption (43), while also binding cholesterol to mitigate hypercholesterolemia (44, 45). Beyond these established metabolic roles, emerging evidence suggests their anti-inflammatory and neuroprotective properties (46). Molecular docking analysis revealed high-affinity interactions between TUDCA/TCA and the bacterial outer membrane protein OmpC (binding energy: −9.4 kcal/mol), with key binding sites localized to conserved domains including PHE-82 and LEU-340 (Fig. 4F). Building upon recent studies highlighting biofilm density as a critical determinant of plasmid conjugation efficiency (39), we hypothesize that TUDCA/TCA accumulation may suppress horizontal dissemination of AMR through dual mechanisms: (i) steric hindrance effects impairing membrane protein functionality, thereby reducing biofilm formation capacity; and (ii) modulation of membrane permeability to limit bacterial fitness. While prior research has documented bile salt-mediated regulation of cholesterol metabolism and inflammation, our work uncovers a novel potential function in controlling AMR propagation. This proposed mechanism requires further validation through targeted experiments, including OmpC knockout models and real-time biofilm dynamics assays, to establish causal relationships between bile acid composition and AMR transmission rates.

The gut microbiota is a complex ecosystem, where probiotics such as *Bacteroides*, *Parabacteroides*, *Lactobacillus*, and *Bifidobacterium* play important roles in maintaining gut health. These probiotics protect the host from pathogenic microorganisms through mechanisms such as competitive inhibition of pathogen growth, modulation of the host’s immune response, and influence on gut barrier function. Emerging evidence highlights the therapeutic potential of next-generation probiotics like *P. goldsteinii*, which secretes anti-inflammatory lipopolysaccharides to mitigate inflammatory responses and demonstrates efficacy against metabolic disorders including obesity (47–51). In this study, the abundance of these probiotics, especially *P. goldsteinii*, in the mouse gut significantly increased after *B. infantis* supplementation, indicating that *B. infantis* can effectively regulate the structure and function of the gut microbiota, enhancing the gut’s defense capabilities. There are complex interactions between bile salts, such as TUDCA and TCA and probiotics. Bile salts can affect the composition and metabolic activity of the gut microbiota, while probiotics can modulate their concentrations and biological activities in the gut through metabolism. With the shift in infant diet, especially after entering the 2–3-year age range, the abundance of *B. infantis* undergoes a precipitous decline (52). Furthermore, even with exogenous supplementation, it has proven difficult to promote its stable re-establishment and colonization (18). Research by Olm et al. highlights significant global geographic disparities in the detection rates of *B. infantis* among infants (53). In non-industrialized nations, the detection rate reaches 90%. In contrast, industrialized countries exhibit a markedly reduced prevalence, with rates approaching zero in some regions. Recent trends associated with global industrialization suggest that infants in highly industrialized areas may risk undetectable levels of *B. infantis* even in early infancy, long before adulthood. Therefore, timely supplementation during early life—particularly during the breast milk or formula feeding stage—to restore its baseline abundance is crucial for infants and holds significant implications for their lifelong microbiota development. In this study, *B. infantis* supplementation not only increased the concentrations of TUDCA and TCA but also promoted the growth of probiotics, suggesting a synergistic effect that may positively impact gut health. For example, TUDCA and TCA can regulate the immune response and inflammatory state of the gut through interactions with the gut microbiota, while probiotics can further enhance their anti-inflammatory and immune-modulating effects by metabolizing these bile salts.

However, this study has several limitations that will be prioritized in subsequent investigations: (i) increasing sample size to enhance statistical power; (ii) extending observation periods to evaluate long-term effects; and (iii) systematically comparing functional heterogeneity across distinct *B. infantis* strains. Notably, our current experimental framework did not assess potential synergistic effects (*in vivo*) between HMO supplementation and *B. infantis* colonization—a critical consideration given HMO’s established role as a growth substrate for this symbiont. Future investigations should also evaluate whether HMO-mediated enhancement of *B. infantis* mucosal adherence and metabolic activity could amplify its microecological modulatory effects, potentially informing synbiotic approaches to optimize probiotic-mediated AMR mitigation strategies. Nevertheless, our findings retain significant translational implications. The work provides novel mechanistic insights into the health-promoting effects of *B. infantis* 15697, demonstrating its capacity to upregulate hepatic CYP7α1-mediated bile acid synthesis and reconfigure commensal metabolic networks via selective enrichment of *P. goldsteinii*. Notably, a positive correlation was identified between *P. goldsteinii* abundance and fecal TUDCA/TCA concentrations. Gut microbiota converts conjugated primary bile acids into secondary bile acids via the enzyme bile salt hydrolase, thereby influencing the intestinal microenvironment and microbial community structure. This conversion process not only facilitates cross-feeding between *P. goldsteinii* and other gut microbes but may also impact the diversity and stability of the gut microbiota, ultimately modulating intestinal health. Moreover, even in the absence of HMO supplementation that could not promote the colonization of *B. infantis*, continuous administration of this strain still effectively regulated bile acid metabolism and reduced the risk of AMR dissemination. This evidence-driven perspective advocates a paradigm shift in combating AMR, transitioning from conventional pathogen-centric approaches to ecological network regulation strategies.

## MATERIALS AND METHODS

### Strains, medium, and chemicals

*E. coli* DH5α, *E. coli* DH5α (pRP4), *E. coli* MG1655::*lacI^q^-pLpp-mCherry-Km*^R^ (pKJK5::*gfpmut3b*) (named *E. coli* MGP) (54), and *B. infantis* 15697 are laboratory-maintained strains. The IncP broad-host range plasmid pRP4 carries genes encoding resistance to tetracycline, ampicillin, and kanamycin. *E. coli* MGP (55) carrying the IncP-1ɛ broad-host range plasmid pKJK5::*gfpmut3b* (56) was used as donors. In this study, Luria-Bertani (LB) medium, *Bifidobacterium* selective medium, de Man, Rogosa, and Sharpe (MRS) agar medium, PYG broth base medium, and *E. coli* color-developing medium were purchased from Hopebio (Qingdao, China), the M9Ca basal medium was purchased from Shenggong (Shanghai, China), and the SIF was purchased from Phygene (Fuzhou, China). The 2FL (cat. no. 41263-94-9) used in this study was purchased from Yuanye (Shanghai, China). The antibiotics used for bacterial cultures in this study were purchased from Solarbio (Beijing, China). Ampicillin, tetracycline, cefoxitin, and kanamycin were dissolved in distilled deionized water (ddH_2_O) at final concentrations of 100, 30, 30, and 50 mg/mL in the medium, respectively. Trimethoprim was dissolved in dimethyl sulfoxide at a final concentration of 30 mg/mL in the medium. Glacial acetic acid was purchased from China National Pharmaceutical Group. All primers used in this study were synthesized with Beijing Tsingke Biotech Co., Ltd.

### Animal management

Firstly, 14 male BALB/c mice weighing 14–18 g were purchased from Pengyue Experimental Animal Breeding Co., Ltd. (Jinan, China) and kept under a 12 h light/12 h dark cycle in standard SPF individually vented cages. The mice were given a common diet and free access to water. Mice aged 3 weeks were randomly divided into experimental groups of seven mice, as follows: control group (gavage with physiological saline [0.9% NaCl] [150 µL] for 12 days and intraperitoneal injection with physiological saline [0.9% NaCl] on the 13th day) (Fig. 1A); *B. infantis* group (gavage with 10^9^ CFU/mL *B. infantis* 15697 [150 µL] for 12 days and intraperitoneal injected physiological saline [0.9% NaCl] on the 13th day) (Fig. 1A). One mouse in the *B. infantis* group was excluded due to a stress event (final *n* = 6). After 4 h of intraperitoneal injection, mice were anesthetized and cervical dislocated. Samples (e.g., liver tissue, ileum tissue, and feces) were taken and stored in 4% paraformaldehyde and liquid nitrogen, respectively.

Second, 16 male BALB/c mice were kept under a 12 h light/12 h dark cycle in standard SPF condition. Mice aged 4 weeks were randomly divided into experimental groups of eight mice, as follows: control group (gavage with physiological saline [0.9% NaCl] [150 µL] for 12 days, gavage with 10^9^ CFU/mL *E. coli* MGP on days 13–15) (Fig. 3A); *B. infantis* group (gavage with 10^9^ CFU/mL *B. infantis* 15697 [150 µL] for 12 days, gavage with 10^9^ CFU/mL *E. coli* MGP [150 µL] on days 13–15) (Fig. 3A).

### Metagenomic sequencing

Individual libraries constructed from each sample were pooled and loaded onto the Illumina HiSeq platform and sequenced using the 2 × 150 bp paired-end read protocol. Raw sequence reads were trimmed using Readfq to remove adapters and low-quality reads. Species annotation was made using Kraken2 (57) and Bracken (58) using clean sequence reads. High-quality reads were assembled into contigs using Metaspades (version 3.15.5) (59). To determine the metagenomic signature of ARGs, open reading frames were predicted using PROKKA (60) and quantified using the Salmon tool (61). To estimate the taxonomic profiles and functional annotations, the gene contig files were annotated using Kraken2 (57), and protein sequences were aligned against the CARD (version 3.2.4) (62) database using DIAMOND (version 2.1.8) (63).

### Targeted metabolomics analysis of fecal bile acid

The bile acid quantification in feces was analyzed by an ultra-performance liquid chromatography tandem mass spectrometry (UPLC-MS/MS) (Acquity UPLC BEH C18, Waters, USA; MS/MS AB 6500 Plus, AB Sciex, USA). Briefly, ileac chyme was ultra-sonically extracted using 400 µL methanol for 30 min and centrifuged with 12,000 rpm for 10 min. The extracts gained from methanol were filtered by 0.22 µm membranes and then quantified bile acids. About 33 bile acids were identified from ileal chyme, and we applied a univariate analysis (*t*-test) to calculate the statistical significance (*P* value).

### mRNA expression by real-time quantitative PCR

Total RNA of liver and ileum was extracted by using FastPure Cell/Tissue Total RNA Isolation Kit V2 (Vazyme, Nanjing, China). Reverse transcription was carried out with 2 mg RNA using M5-Super qPCR RT Kit with gDNA remover (Vazyme, Nanjing, China) to synthesize the cDNA. The ileal expression of *Tgr5* and hepatic mRNA expression of *Cyp7α1*, *Fxr*, and *Tlr4* were determined by SYBR green real-time PCR using a StepOne Plus real-time PCR system (Applied Biosystems, USA). β-Actin was used as an internal standard for normalization. *E. coli* DH5α (pRP4) was cultured in liquid LB medium with pH values of 5.5 and 7.0, respectively, at 37°C until the OD_600_ value reached between 0.4 and 0.6. Total RNA was extracted using FastPure Cell/Tissue Total RNA Isolation Kit V2 (Vazyme, Nanjing, China), following the instructions provided. Subsequent operations were carried out as described above, with 16S rRNA serving as the internal standard for normalization. The primer sequences are shown in Table S2.

### Fluorescence imaging

Starting on day 13, two infected mice (killed after isoflurane anesthesia) in each group were randomly picked and monitored every day using a standard set of exposure times in an IVIS Lumina II (Perkin Elmer). The dynamics of AMR dissemination were observed. The *E. coli* MGP strain expresses red fluorescence, whereas green fluorescence appears when the pKJK5::*gfpmut3b* plasmid is horizontally transferred from *E. coli* MGP into the enteric commensal bacteria. Fluorescence was quantified with Living Image version .4.2 software (Perkin Elmer) and corrected for background fluorescence.

### Determination of bacterial growth and metabolite pH

The *B. infantis* 15697 frozen stock tube was taken out from the −80°C refrigerator and quickly thawed in a 37°C constant temperature water bath. In the anaerobic sterile glove box, 0.2 mL of liquid was taken from the frozen stock tube with a pipette and vertically dropped onto the center of the PYG agar plate (no need to spread with a spreader). The plate was placed upright in the anaerobic incubator and cultured at 37°C for 24–36 h. Single colonies on the plate were inoculated into sterile oxygen-free serum bottles containing PYG liquid medium with a sterile inoculating loop and cultured at 37°C for 44–48 h. The resulting bacterial suspension was used as the seed liquid for subsequent experiments. The ratio of seed liquid to test medium was 1:9, that is, 2 mL of seed liquid was inoculated into 18 mL of test medium. The experimental group with HMO contained 2FL at a final concentration of 1.2 g/L. Each group of bacteria was statically cultured in a 37°C anaerobic glove box, and the pH of the culture medium of each group was measured with a pH meter every 24 h. The optical density of the culture medium at 600 nm was measured in the early and late stages of cultivation (this operation was designed with three technical parallels).

### Determination of biofilm formation

*E. coli* cell cultures were processed according to a previous research method (64), and then absorbance at 590 nm was measured to quantify the quality of the biofilm. The negative control (OD590_negative_) was the double absorbance value of the sterile medium. All experiments were performed at least six times.

### Molecular docking analysis

To evaluate the binding energy and interaction patterns between candidate drugs and their targets, we employed Autodock Vina 1.2.2, a computer-based protein-ligand docking software. First, we obtained the X-ray structure of OmpC (PDB ID: 2J1N, resolution: 2.00 Å) from the Protein Data Bank (PDB). Next, we retrieved the 3D structures of TCA (Compound CID: 6675) and TUDCA (Compound CID: 2733768) in SDF format from the PubChem database. We prepared the protein and ligand files by converting all protein and molecular files into PDBQT format and adding polar hydrogen atoms. The docking pocket was set as a rectangular box measuring 66 Å × 84 Å × 78 Å with a grid spacing of 0.05 nm. To assess the affinity of candidate drugs for their targets, we conducted molecular docking analysis. Autodock Vina 1.2.2 was used to obtain the binding poses of candidate drugs with the protein and the binding energy of the best protein-ligand complex interactions. We systematically analyzed the binding interface of the protein-ligand complex using PLIP and LigPlus and supplemented interaction details using PyMOL 2.5 software.

### Statistical analysis

The statistical tests used are indicated in the figure legends. The statistically significant differential species were evaluated by the LEfSe analysis (phyloseq, 1.44.0), ANCOM-BC (ANCOMBC, 2.8.1), and MAasLin2 (Maaslin2, 1.15.1) in R. Correlation analysis between differential bile acids and species level of microbiota was investigated by Pearson analysis and mental test (ggcor) using R. *P* values <0.05 were considered statistically significant: *, *P* < 0.05; **, *P* < 0.01; ***, *P* < 0.001.

## Data Availability

The raw metagenomics data are publicly available from the National Center for Biotechnology Information (nih.gov) Sequence Read Archive (SRA) under the Bioproject accession number: PRJNA1247700.

## SUPPLEMENTAL MATERIAL

Supplemental Figures msystems.00728-25-s0001.pdf Figures S1-S6.

Table S1 msystems.00728-25-s0002.xlsx The content of bile acids in the samples.

Table S2 msystems.00728-25-s0003.docx Primer sequences used in this study.

ASM does not own the copyrights to Supplemental Material that may be linked to, or accessed through, an article. The authors have granted ASM a non-exclusive, world-wide license to publish the Supplemental Material files. Please contact the corresponding author directly for reuse.
