# Supplementary material for: Bifidobacterium infantis modulates intestinal microecology to inhibit the spread of antimicrobial resistance
Source: mSystems. 2025 Oct 31;10(11):e00728-25. doi: 10.1128/msystems.00728-25 (PMC12625698; doi:10.1128/msystems.00728-25)

Supplementary File_ Figure S1-S6

Content

Figure S1··2

Figure S2··3

Figure S3··4

Figure S4··5

Figure S5··6

Figure S6··7

Figure S1. Expression levels of relevant genes in liver tissue.

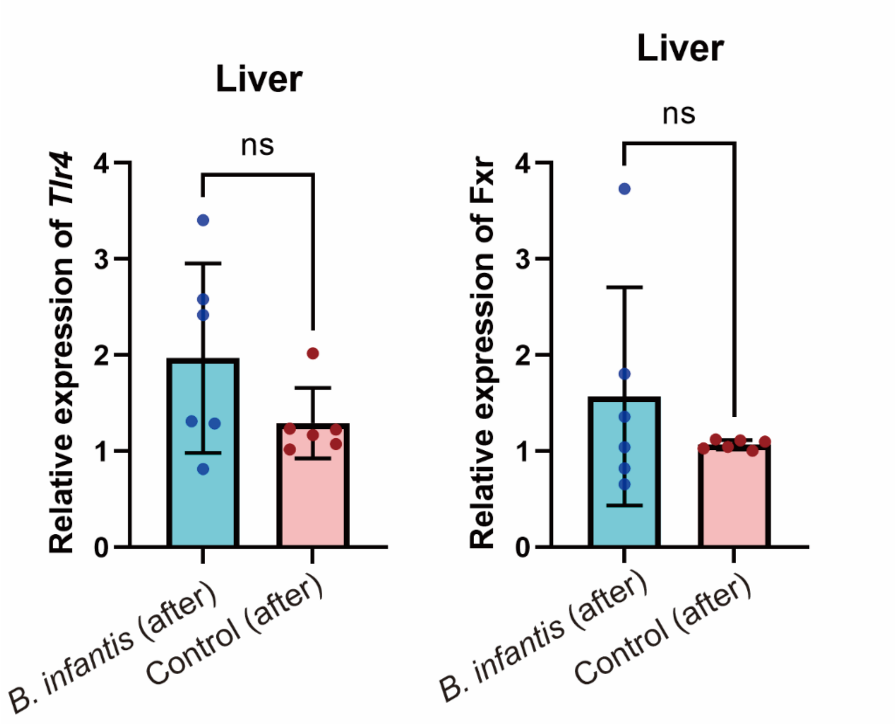

Figure S2. The α- and β-diversity of the gut microbiota in mice. A) α-diversity index; B_a: *B. infantis*_after, B_b: *B. infantis*_before, C_a: Control_after, C_b: Control_before; B) PCoA analysis.

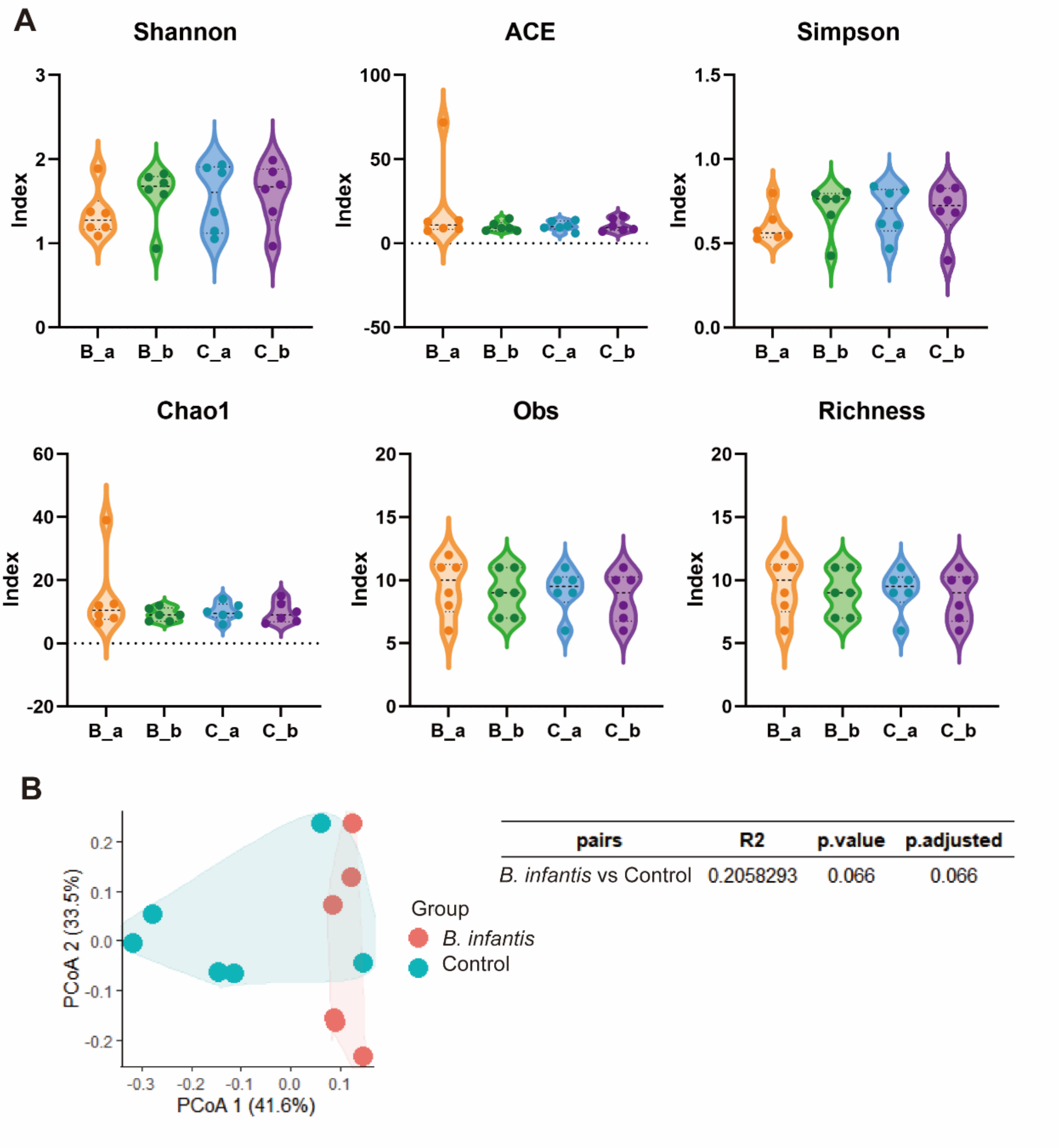

Figure S3. LEfSe analysis (the *p*-values were not corrected).

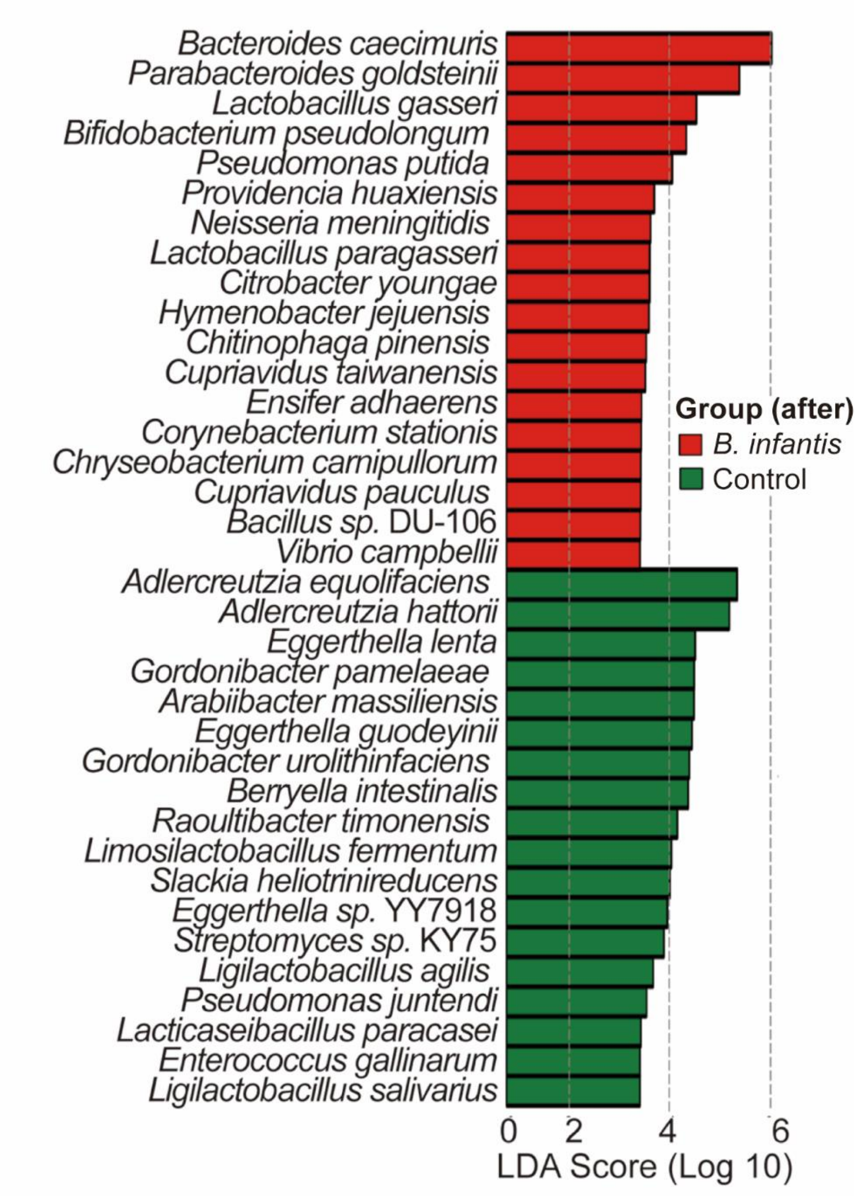

Figure S4. Relative abundance of enriched strains in the *B. infantis* 15697 gavage group (FDR-corrected p-values > 0.05).

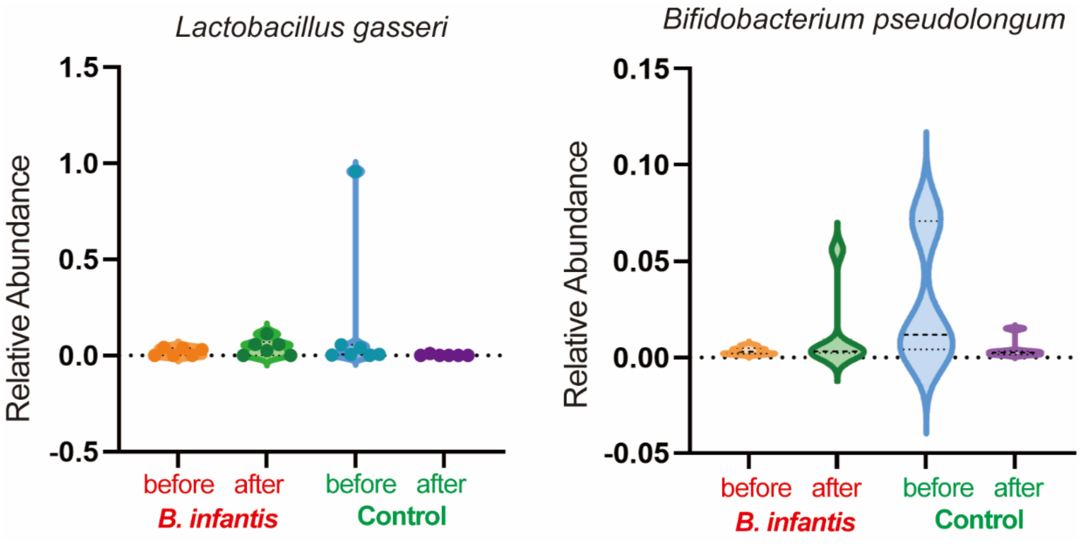

Figure S5. The association analysis between strains and ARGs before and after gavage in the control group and *B. infantis* group.

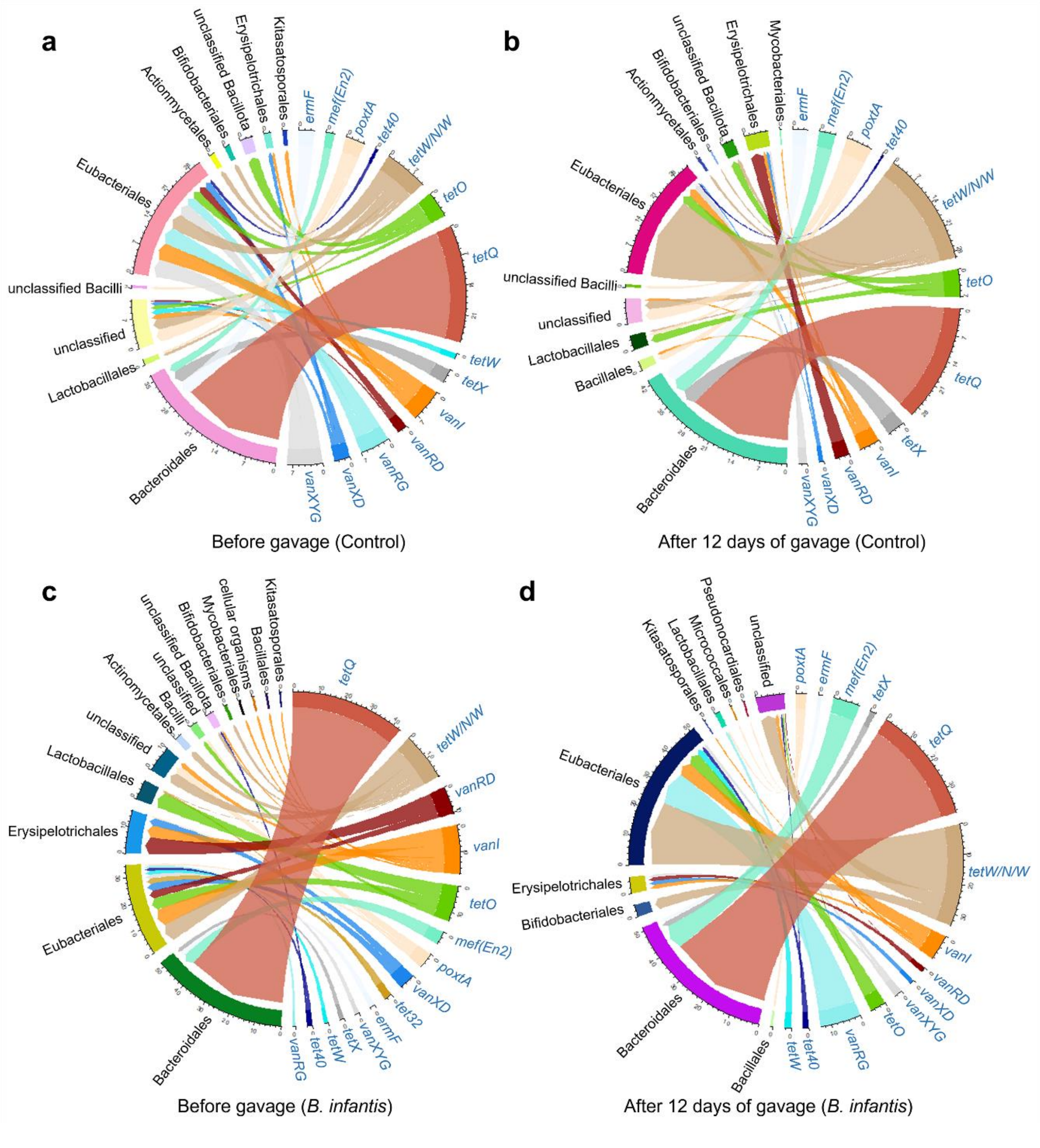

Figure S6. The association analysis between strains and tetracycline resistance-associated mARGs before and after gavage in the control group.

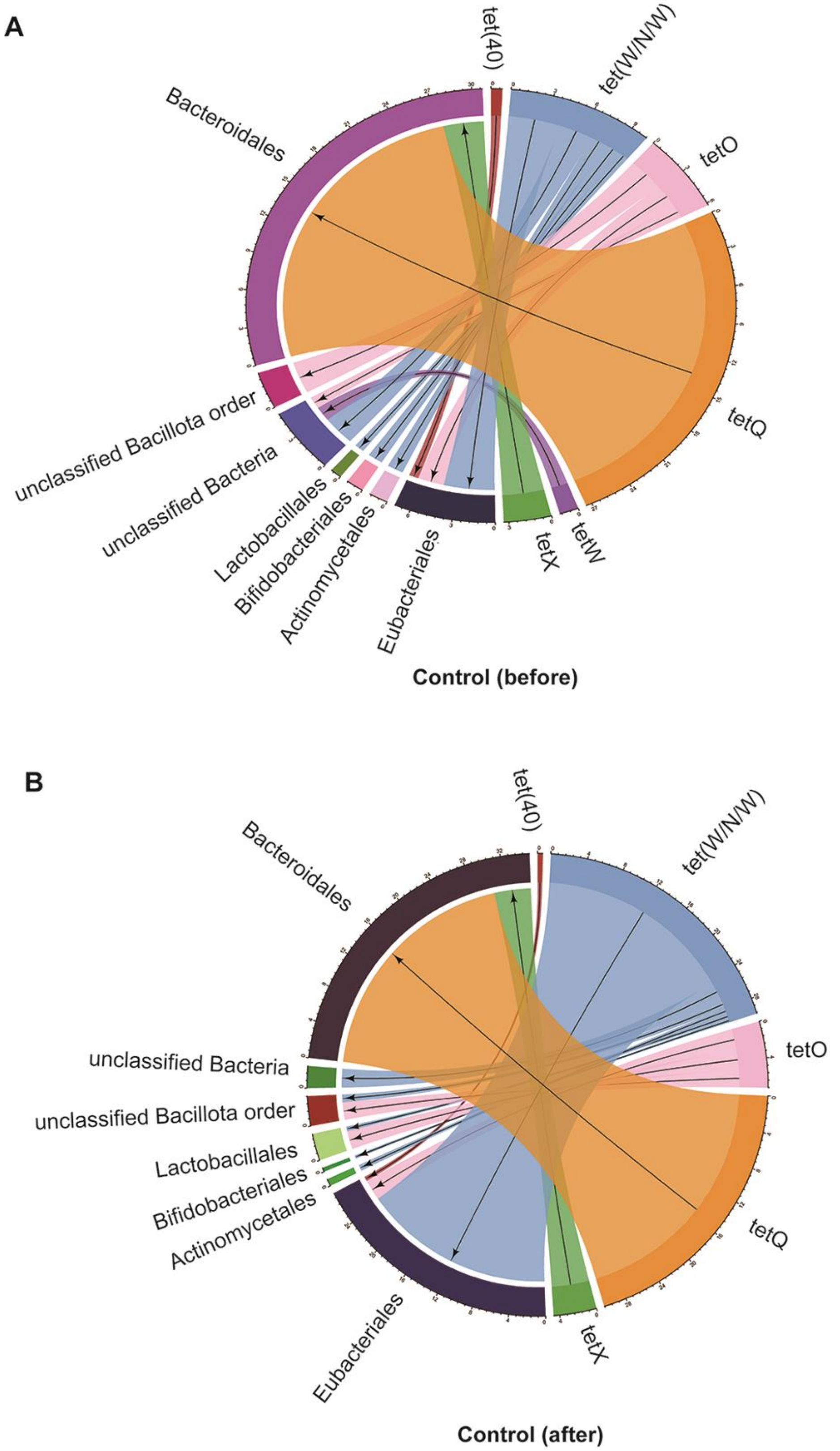

Supplement: Supplemental Figures — Figures S1-S6. [file msystems.00728-25-s0001.pdf]
