# Supplementary material for: Bifidobacterium infantis modulates intestinal microecology to inhibit the spread of antimicrobial resistance
Source: mSystems. 2025 Oct 31;10(11):e00728-25. doi: 10.1128/msystems.00728-25 (PMC12625698; doi:10.1128/msystems.00728-25)
Supplement: Table S2 — Primer sequences used in this study. [file msystems.00728-25-s0003.docx]

**Table S2. Primer sequences used in this study.**

| Gene | Primer | Sequence (5’ to 3’) | Tm (℃) | Reference |
| --- | --- | --- | --- | --- |
| *Cyp7α1* | F | TCAACGATACACTCTCCACC | 60 | (1) |
|  | R | TTCATTGCTTCAGGGCTCC |  |  |
| *Tgr5* | F | GTTATCGCTCATCTCATTGGG | 60 | (1) |
|  | R | GATTGTCCCTCTTGGCTCT |  |  |
| *Fxr* | F | TTACAGGCTACGGACGAGT | 60 | (1) |
|  | R | CTTGAGGAAACGGGACATTG |  |  |
| *Tlr4* | F | TTCAGAGCCGTTGGTGTATC | 60 | (1) |
|  | R | CCCATTCCAGGTAGGTGTTT |  |  |
| β-actin | F | GGCTGTATTCCCCTCCATCG | 60 | (1) |
|  | R | CCAGTTGGTAACAATGCCATGT |  |  |
| *ompC* | F | CCTACATGCGTCTTGGCTTC | 60 | This study |
|  | R | CACCGAATTCTGGCAGTACG |  |  |
| *acrA* | F | AAGCAGGTGTCTCTCTCTAT | 60 | This study |
|  | R | TACCGAGCAGTTTCTGATAA |  |  |
| *tolC* | F | GTTCTGGCCCATATTGCTATC | 60 | This study |
|  | R | CGGAATTTCTGACACCTCTTAC |  |  |
| *ybhF* | F | CGCTGTCGGGTTTCAGTAAT | 60 | This study |
|  | R | GTACCATTCACGCCGGTTAT |  |  |
| *16S rRNA* | F | CCCAGATGGGATTAGCTTGT | 60 | (2) |
|  | R | TCTGGACCGTGTCTCAGTTC |  |  |

**Reference**

1. Lin Z, Wu J, Wang J, Levesque CL, Ma X. 2023. Dietary *Lactobacillus reuteri* prevent from inflammation mediated apoptosis of liver via improving intestinal microbiota and bile acid metabolism. Food Chem 404:134643.

2. Kim JY, Lee J-L. 2014. Multipurpose assessment for the quantification of Vibrio spp. and total bacteria in fish and seawater using multiplex real-time polymerase chain reaction. J Sci Food Agric 94:2807–2817.
